# Supplementary material for: Antioxidant Enzymatic Activities and Growth Response of Quinoa (Chenopodium quinoa Willd) to Exogenous Selenium Application
Source: Plants (Basel). 2021 Apr 7;10(4):719. doi: 10.3390/plants10040719 (PMC8068041; doi:10.3390/plants10040719)
Supplement: Supplementary file 1 [file plants-10-00719-s001.pdf]

plants

MDPI

Supplementary figures

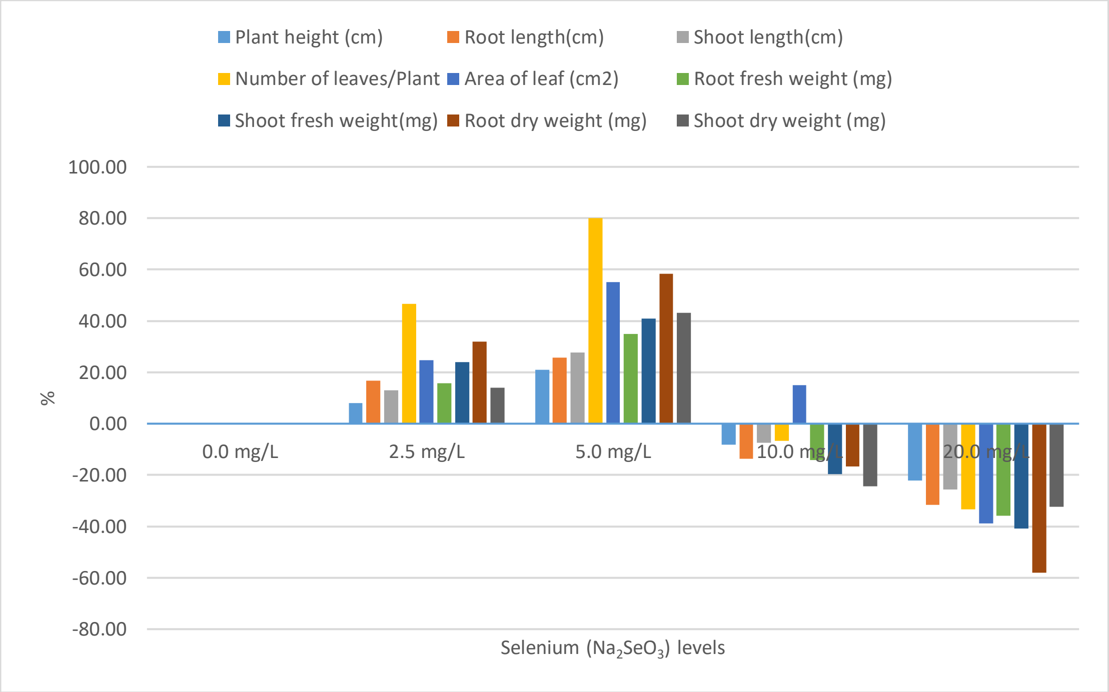

Figure S1. Relative increasing or decreasing in the growth parameters under various selenium levels (Na_2SeO_3) compared with quinoa plants' control treatment after 30 days of treatment. Comparisons of means for different selenium levels (Na_2SeO_3) at Alpha 0.05, Standard error for: plant height = 3.64, Root length = 3.10, shoot length = 3.20, number of leaves/ plants = 4.10, Area of leaf = 3.60, root fresh weight = 3.20, shoot fresh weight = 3.30, root dry weight = 3.53 and shoot dry weight = 3.33.–

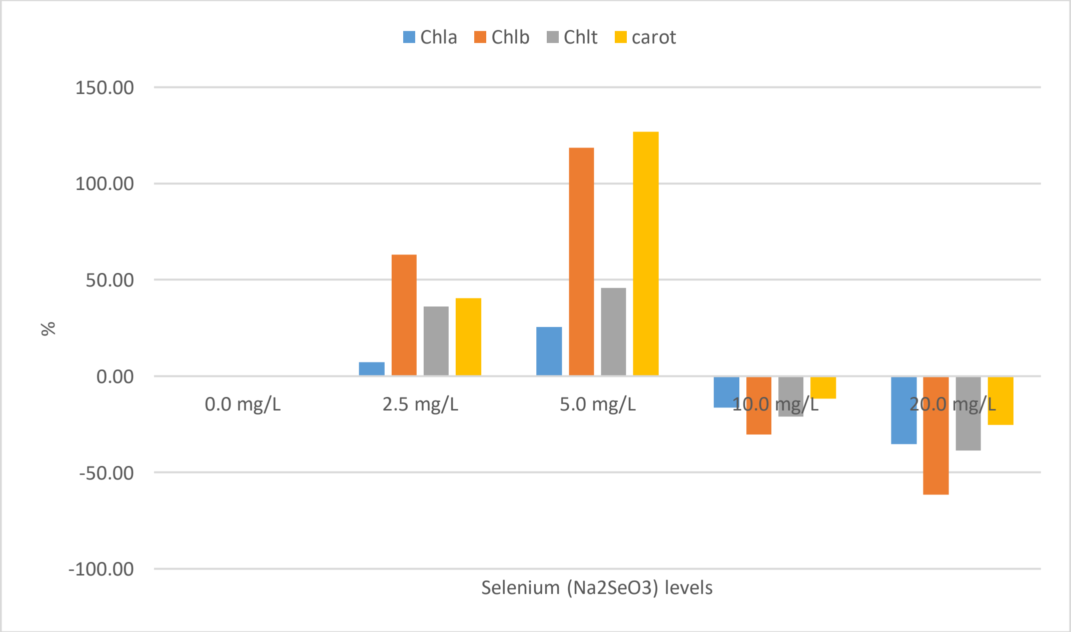

Figure S2. Relative increasing or decreasing in the photosynthetic pigments contents under various selenium levels (Na_2SeO_3) compare with quinoa plants' control treatment after 30 days of treatment. Comparisons of means for different selenium levels (Na_2SeO_3) at Alpha 0.05, the standard error for chlorophyll-a = 2.01, chlorophyll-b = 2.74, total chlorophyll = 2.21 and carotenoids = 3.01.

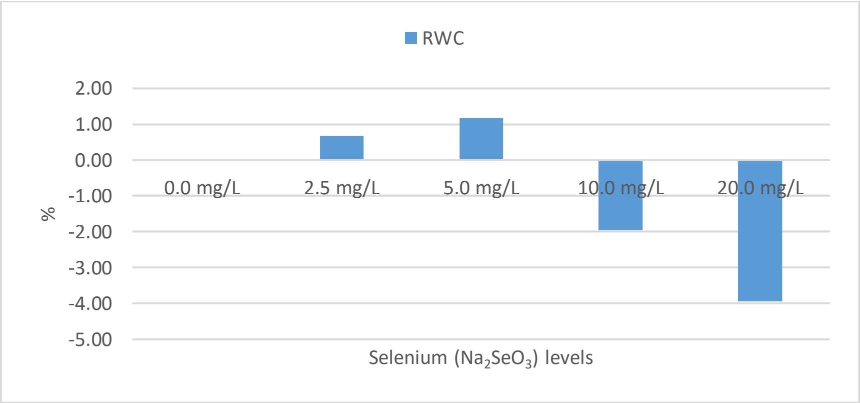

Figure S3. Relative increasing or decreasing in the relative water contents under various levels of selenium (Na_2SeO_3) compare with the control treatment of quinoa plants after 30 days of treatment. Comparisons of means for different selenium levels (Na_2SeO_3) at Alpha 0.05, the standard error for RWC = 1.11.

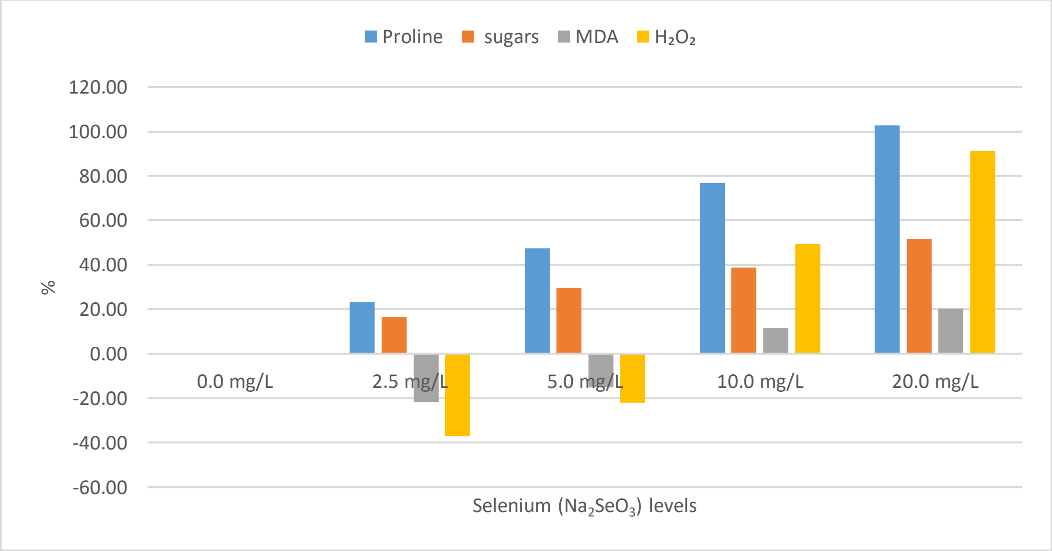

Figure S4. The relative increase in the proline and total soluble sugars under various levels of selenium (Na_2SeO_3) compare with the control treatment of quinoa plants after 30 days. Comparisons of means for different selenium levels (Na_2SeO_3) at Alpha 0.05, the standard error for proline = 4.01 and total soluble sugar = 3.20, MDA = 2.42 and H_2O_2 = 3.46.

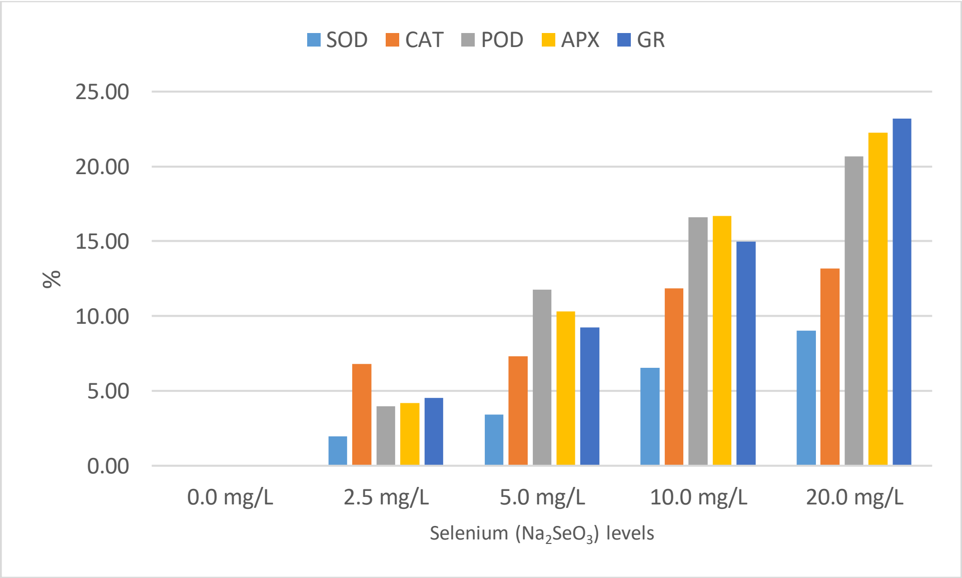

Figure S5. The relative increase in antioxidant enzyme activity under various levels of selenium (Na_2SeO_3) compare with the control treatment of quinoa plants after 30 days. Comparisons of means for different selenium levels (Na_2SeO_3) at Alpha 0.05, the standard error for SOD = 2.40, CAT = 2.50, POD = 2.61, APX = 2.62 and GR = 2.62.
